# FACTORS ASSOCIATED WITH PARTICIPATION IN FUNCTION-FOCUSED CARE AMONG HOSPITALIZED PATIENTS LIVING WITH DEMENTIA

**DOI:** 10.1093/geroni/igad104.0703

**Published:** 2023-12-21

**Authors:** Ashley Kuzmik

**Affiliations:** Pennsylvania State University, State College, Pennsylvania, United States

## Abstract

Function focused care is an approach used to increase physical activity in hospitalized older adults living with dementia. This study tested the factors associated with participating in function focused care among patients living with dementia. This was a cross-sectional descriptive study using baseline data from the first 365 participants from the first 10 hospitals in the study entitled, Testing the Effectiveness of Function Focused Care for Acute Care Using the Evidence Integration Triangle. Structural equation modeling was used for model testing. The mean age of the participants was 83.2 (SD=8.0) and the majority were women (64%) and white (69%). Fifteen of the 27 hypothesized paths were significant and explained 25% of the variance in performance of function focused care. Cognition, quality of care interactions, behavioral and psychological symptoms associated with dementia, physical resilience, and pain were all indirectly associated with performance of function focused care through function and/or pain. Tethers, function, and quality of care interactions were all directly associated with function focused care. The x2 / df was 5.37, the normed fit index was .87 and the Root Mean Error Square of Approximation was .12. For hospitalized patients living with dementia the focus of care should be on treating pain and behavioral symptoms, decreasing tethers, and improving quality of care interactions so as to optimize resilience, function and participation in function focused care.

